# 1030. Epidemiology and Microbiologic Characteristics of Post-operative Central Nervous System Infections following Endoscopic Endonasal Surgery

**DOI:** 10.1093/ofid/ofac492.871

**Published:** 2022-12-15

**Authors:** Sunish Shah, Joseph Durkin, Karin E Byers, Carl H Snyderman, Paul A Gardner, Ryan K Shields

**Affiliations:** Antibiotic Management Program, UPMC Presbyterian Hospital, Pittsburgh, PA, Pittsburgh, Pennsylvania; Department of Pharmacy, UPMC Presbyterian Hospital, Pittsburgh, PA, Pittsburgh, Pennsylvania; Division of Infectious Diseases, University of Pittsburgh, Pittsburgh, Pennsylvania, USA., Pittsburgh, Pennsylvania; Department of Otolaryngology, University of Pittsburgh, Pittsburgh, USA, Pittsburgh, Pennsylvania; Department of Neurosurgery, University of Pittsburgh, Pittsburgh, PA, Pittsburgh, Pennsylvania; University of Pittsburgh, Pittsburgh, Pennsylvania

## Abstract

**Background:**

Endoscopic Endonasal Surgery (EES) is an innovative surgical technique to remove brain tumors and lesions. Post-operative central nervous system (CNS) infections following EES are poorly described. The objective of this study was to define the epidemiology and characteristics of post-EES CNS infections.

**Methods:**

Adult patients who underwent EES between 1/2010 and 7/2021 were evaluated and included if microbiologically confirmed CNS infection occurred within 30 days of EES. Suspected contaminants, ventricular drain colonization, and pre-EES CNS infections were excluded.

**Results:**

Overall, 2005 patients underwent EES; 1.8% (37/2005) developed CNS infection. The median [IQR] age was 51 [42-60] years, 32.4% (12/37) were female, and 54% (20/37) had a prior EES. The most common indications for EES were tumor resection [67.6% (25/37)] and cerebrospinal fluid (CSF) leak repair [24.3% (9/37)]. Post-operative CSF leaks were documented in 70.3% (26/37) of patients and 24.3% (9/37) had an extra-ventricular drain or shunt in place for >48 hours at the time of infection. Ceftriaxone prophylaxis was prescribed in 64.9% (24/37) of cases and other regimens varied. The median [IQR] time from EES to diagnosis of CNS infection was 12 [6-19] days. The most common pathogens were *S. aureus, Enterobacterales,* and *P. aeruginosa***(Fig 1)**. Among 20 patients with prior EES, pathogens included *S. aureus* (5/20), *Enterobacterales* (3/20), *Enterococcus spp.* (3/20) and polymicrobic infections (3/20). Overall, 35.1% (13/37) of patients developed CNS infection due to a pathogen susceptible to pre-EES prophylaxis. Among those colonized with MRSA at time of EES, 75% (3/4) developed MRSA CNS infection compared to 6.1% (2/33) of non-colonized MRSA patients (P=0.005). The overall 30-day mortality rate was 2.7% (1/37).

**Figure:**
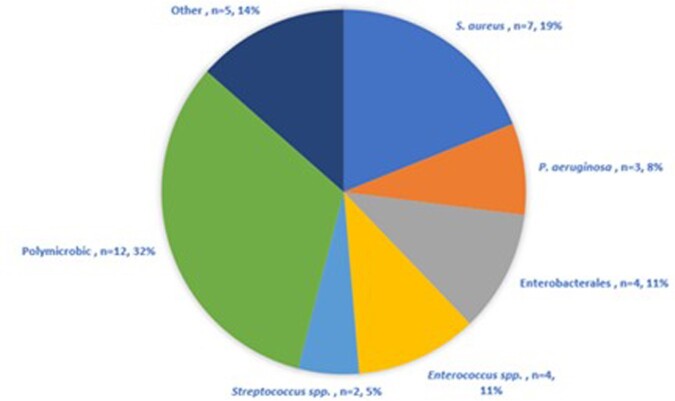
Microbiology

A polymicrobic case was defined as >1 pathogen isolated from CSF (n=1) or from rhinocerebral tissue if CSF cultures were negative (n=11). Among polymicrobic cases (n=12), P. aeruginosa (n=5), Enterococcus spp. (n=4). and S. aureus (n=3) were predominant. Cases labeled as other consisted of Trichoderma spp, A. xylosoxidans, P. acnes, S. epidermidis, Peptostreptococcus spp.

**Conclusion:**

CNS infection post-EES is rare and causative pathogens vary. Given the predominance of *S. aureus*, antimicrobial prophylaxis should ensure adequate coverage of this pathogen in addition to sinus flora, and programs may benefit from screening patients for MRSA colonization pre-EES. Our data also suggest that prophylaxis should target Gram-negative and other colonizing bacteria among patients with prior EES.

**Disclosures:**

**Ryan K. Shields, PharmD, MS**, Infectious Disease Connect: Advisor/Consultant|Merck: Advisor/Consultant|Merck: Grant/Research Support|Roche: Grant/Research Support.

